# Novel technique to suppress hydrocarbon contamination for high accuracy determination of carbon content in steel by FE-EPMA

**DOI:** 10.1038/srep29825

**Published:** 2016-07-19

**Authors:** Takako Yamashita, Yuji Tanaka, Masayasu Yagoshi, Kiyohito Ishida

**Affiliations:** 1 JFE Steel Corporation, Steel Research Laboratory, 1 Kawasaki-cho, Cho-ku, Chiba 260-0835, Japan; 2 JFE Steel Corporation, Steel Research Laboratory, 1-1, Minamiwatarida-cho, Kawasaki-ku, Kawasaki, 210-0855, Japan; 3 Tohoku University, 6-6-02, Aramaki-aza Aoba, Aoba-ku, Sendai 980-8579, Japan

## Abstract

In multiphase steels, control of the carbon contents in the respective phases is the most important factor in alloy design for achieving high strength and high ductility. However, it is unusually difficult to determine the carbon contents in multiphase structures with high accuracy by electron probe microanalysis (EPMA) due to the unavoidable effect of hydrocarbon contamination during measurements. We have investigated new methods for suppressing hydrocarbon contamination during field emission (FE) EPMA measurements as well as a conventional liquid nitrogen trap. Plasma cleaner inside the specimen chamber results in a improvement of carbon-content determination by point analysis, increasing precision tenfold from the previous 0.1 mass%C to 0.01 mass%C. Stage heating at about 100 °C dramatically suppresses contamination growth during continuous point measurement and mapping. By the combination of above two techniques, we successfully visualized the two-dimensional carbon distribution in a dual-phase steel. It was also noted that the carbon concentrations at the ferrite/martensite interfaces were not the same across all interfaces, and local variation was observed. The developed technique is expected to be a powerful tool for understanding the mechanisms of mechanical properties and microstructural evolution, thereby contributing to the design of new steel products with superior properties.

In recent years, a combination of higher strength and improved ductility has been demanded in high strength automotive steels. These properties are achieved by using hard phases such as martensite and bainite to increase strength and strain-induced transformation of the retained austenite phase to improve elongation. Carbon is the most important element in controlling the microstructure of steel, including the ferrite, austenite^1^,^2^, bainite^3^,^4^ and martensite phases, which in turn determines the mechanical properties of steel products^5^,^6^. In multiphase steels, control of the carbon contents in these phases is a crucial factor in alloy design for achieving high strength simultaneously with high ductility. In order to clarify the mechanisms responsible for the formation of martensite, bainite and retained austenite^7^,^8^, it is important to understand the relationship between the microstructure and carbon distribution with high accuracy and high spatial resolution^9^.

The key requirements for dual-phase (DP) steels are low yield strength, high tensile strength and good elongation^10^,^11^. The carbon distribution in DP steels has been investigated by field-emission electron probe microanalysis (FE-EPMA)^12^,^13^, and the carbon distributions corresponding to the ferrite and martensite phases were clarified by FE-EPMA line analysis.

Recently, the ultimate mechanical properties of the steel material itself have been pursued by utilizing thermomechanical controlled processing (TMCP). As part of this effort, fundamental research on a multi-cycle annealing process has been carried out, focusing not only on refinement of hard second phases such as martensite or bainite, but also on the fine distribution of retained austenite as an approach to improving elongation. However, it was difficult to understand the carbon distributions of these newly-developed materials from the FE-EPMA line analyses in previous papers^12^,^13^. Since these high strength steels are characterized by a fine, heterogeneous microstructure, a two-dimensional analysis technique with higher accuracy and spatial resolution is needed for analysis of their phase transformation phenomena.

EPMA measurement of carbon concentration is used in conventional quantitative analyses of steel, but it is nevertheless a problematic technique due to the buildup of hydrocarbon contamination on the sample surface during measurement^14^,^15^,^16^. Because this type of contamination is caused by interaction between hydrocarbon contaminants on and around the sample and the incident electron beam^14^, efforts to improve the carbon measured by using several techniques aiming to reduce hydrocarbon contamination. Heavy contamination remaining on the sample surface is removed by preprocessing such as polishing and rinsing, and the buildup of hydrocarbon contaminations during measurement is reduced by installing a liquid nitrogen cold trap^17^ immediately above the sample in the specimen chamber. Although the liquid nitrogen cold trap is an effective tool for decreasing contamination^17^,^18^, the residual carbon intensity is not negligible, resulting in a high background. Other anti-contamination methods have also been developed, including oxygen air-jet^19^,^20^ and plasma cleaning^21^,^22^ techniques, but their best performance is limited to line analysis with an accuracy of 0.02 mass% and precision of 0.05 mass% by FE-EPMA^13^.

The aim of our research was to develop a new FE-EPMA technique which greatly reduces hydrocarbon contamination beyond the levels achieved in previous studies. To this end, we developed a new FE-EPMA microprobe instrument incorporating the following three types of anti-contamination devices: (1) plasma cleaners in the specimen chamber and the preparation chamber, (2) a heating-type sample stage and (3) three carbon analyzers. This paper presents quantitative point analysis and quantitative two-dimensional analysis data obtained with the newly-developed instrument and discusses these results from the viewpoint of anti-contamination performance.

## Method

### Field emission electron microprobe instrument

In carbon microanalysis to examine the microstructure of steel, the measurement method must be nondestructive and must have an analysis area smaller than the crystal grain size. FE-EPMA satisfies both of these requirements, as it is a nondestructive technique and the electron beam secures the requisite small analysis area.

In this work, we developed a new FE-EPMA instrument equipped with a combination of anti-contamination devices to further reduce hydrocarbon contamination. The basic instrument was a JEOL JXA-8530F (JEOL Ltd., Tokyo, Japan), in which we installed (1) plasma cleaners in the specimen chamber and preparation chamber, (2) a heating-type sample stage in the specimen chamber and (3) three carbon analyzers. The new instrument is also equipped with a conventional liquid nitrogen trap.

Although contamination removal by plasma irradiation has previously been demonstrated and has a record of success in scanning electron microscopy^23^,^24^, it has not been used in the EPMA sample chamber because the plasma would damage the organic membrane window at the detector tip (private communication). To avoid this problem, we placed retractable protective plates in front of all the detectors for protection from plasma irradiation. A high-vacuum type plasma cleaner (Plasma Asher GV10x, ibss Ltd., CA, USA) was used in this study. This is a suitable anti-contamination device for the EPMA specimen chamber because it operates under a rather high vacuum and generates a directional flow of plasma to the specimen.

The second technique is a heating-type sample stage for the FE-EPMA. Using a ceramic heater, we devised an original heating stage which operates at around 100 °C during EPMA measurements.

Finally, in order to increase the total X-ray signal intensity from the carbon in steel, we installed three high efficiency carbon analyzers (analyzing crystal: LDE6H).

**Figure 1:**
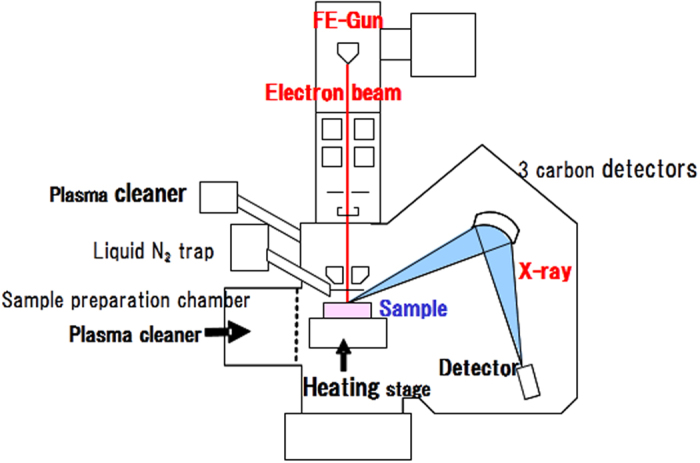
Schematic diagram of developed instrument for carbon mapping in steels.

Figure 1 shows an overview of the contamination removal techniques in the developed instrument.

### Specimens

Pure Fe and reference steels with carbon contents from 0.089 mass% to 0.680 mass% (Certified reference materials, AIST^25^) were used to investigate the analytical performance of the developed instrument. Specimens cut from cold-rolled sheets of a DP steel (Fe-0.15 mass%C-2.0 mass%Si-1.5 mass%Mn) were intercritically annealed at 750 °C for 150 s in a salt bath furnace, followed by water quenching. The specimen surfaces were mechanically polished up to 0.05 μm alumina suspensions to obtain flat surfaces with minimum surface relief, and the specimen was cleaned in an ultrasonic bath with ethanol and dried with hot air. The two-dimensional carbon distribution of the steel was then measured with the developed instrument.

### FE-EPMA measurements

All specimens were treated by the plasma cleaner (Evactron CombiClean, XEI Scientific Inc., CA, USA) in the preparation chamber^22^ before they were introduced into the specimen chamber. Depending on the purpose, some or all of the anti-contamination devices were used before or during the measurements. The primary electron beam accelerating voltage was set to 7 kV to obtain the optimum balance of spatial resolution and signal intensity. This condition is advantageous for imaging and 2-dimensional analysis due to its higher spatial resolution. The beam current and primary electron beam diameter were set to 50 nA and spot (minimum) to 50 μm, respectively. All measurements were performed under a vacuum of 1.0E-4 Pa order.

## Results and Discussion

**Figure 2:**
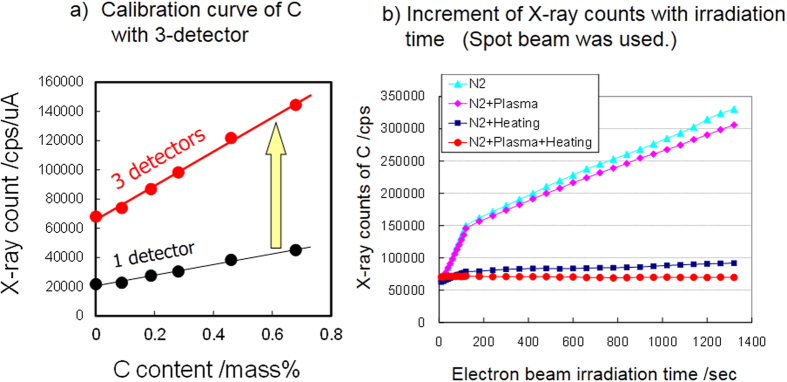
Contamination reduction function of developed instrument. (**a**) Calibration curve of C with 1 or 3 detectors. (**b**) Increment of X-ray counts with electron beam irradiation time.

Figure 2a shows the carbon K (C–K) X-ray intensities measured by one and three spectrometers as a function of the carbon content for pure Fe and the five reference steels. A primary electron beam diameter of 50 μm was used in this experiment. The intensities measured by the three spectrometers are significantly higher than those measured by one spectrometer. This indicates that statistical error is reduced by a factor of about 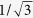 compared to conventional instruments. The change of C–K X-ray intensity of pure Fe measured with a focused primary electron beam is plotted as a function of beam irradiation time in Fig. 2b. The dwell time was 10 s or 60 s. The interval was a constant 10 s for every point, after which the electron beam was turned off. The X-ray counts from the three detectors were integrated. Figure 2b shows the results obtained with several combinations of anti-contamination devices. With the liquid nitrogen trap alone (corresponding to the conventional instrument; cyan diamond), X-ray intensity increases linearly with increasing electron beam irradiation time. Because pure Fe contains no carbon, this increase of X-ray intensity corresponds to the increase of hydrocarbon contamination during the measurement. The change in the slope at around 160 s is due to the effect of the irradiation time, as the electron beam irradiation time was changed from 10 s to 60 s in this measurement. The results when the plasma cleaner was applied in the specimen chamber before the measurement are shown in Fig. 2b as ♦. The working time of plasma cleaning was 5 min. The C–K X-ray intensity under this experimental condition is slightly lower than that with the liquid nitrogen trap alone, showing that the contamination reduction effect of plasma irradiation is small. However, the C–K X-ray intensity is drastically reduced by using the heating stage. The specimen was heated at about 100 °C during the measurement (blue squares). When plasma irradiation in the specimen chamber is used in combination with heating (red circles), the X-ray intensity becomes weaker and is almost constant up to 1300 sec of electron irradiation time. 1300 sec were accumulated time for irradiation of electron beam.

These results show that sample heating has an extremely large anti-contamination effect and can suppress contamination during measurement to a degree that had not previously been achieved. While preheating at 300 °C has been attempted as an anti-contamination measure for transmission electron microscopy (TEM)^26^, very few studies have examined the effects of low-temperature heating on hydrocarbon contamination on metal surfaces. Although the mechanism responsible for suppressing hydrocarbon buildup when low-temperature heating is applied in the EPMA is still unclear, we speculate that the contaminant molecules on the sample surface are forcefully desorbed by heat, undergo surface and/or gas-phase diffusion, and thus are unavailable at the electron beam irradiation site. Further research will be needed to clarify the anti-contamination effect of low-temperature heating during EPMA measurements, but sampling heating is clearly an effective technique for preventing contamination buildup.

The C–K X-ray intensity measured by our instrument displays a good correlation with the C content in the steel specimens, as shown in Fig. 2a. Measurements of the C–K X-ray intensity for pure Fe and the five reference steels using the liquid nitrogen trap and plasma cleaner in the specimen chamber were performed a total of 13 times. Each measurement was carried out from the sample polishing step on different days. The beam diameter was a 5 μm spot. The dwell time for each measurement was 10 s, and the X-ray counts from the three detectors were integrated. Typically, the X-ray intensity of about 70,000 cps/μA was obtained for the pure Fe specimen.

The uncertainties of EPMA measurements are discussed in detail by Pinard, P.T. *et al*.^13^ and Marinenko, R.B. *et al*.^27^. Pinard obtained an accuracy of 0.02 mass% C and a precision better than 0.05 mass% C for carbon line analysis^13^. Liu, Z.-Q. *et al*.^12^ obtained a typical standard error of 0.03 mass%, and Duerr, J.S. *et al*.^28^ proved that this technique is well suited for measurements of C contents below 0.1 mass%. The standard deviation and accuracy of the carbon content measurement by our instrument are discussed in the next paragraphs.

**Figure 3:**
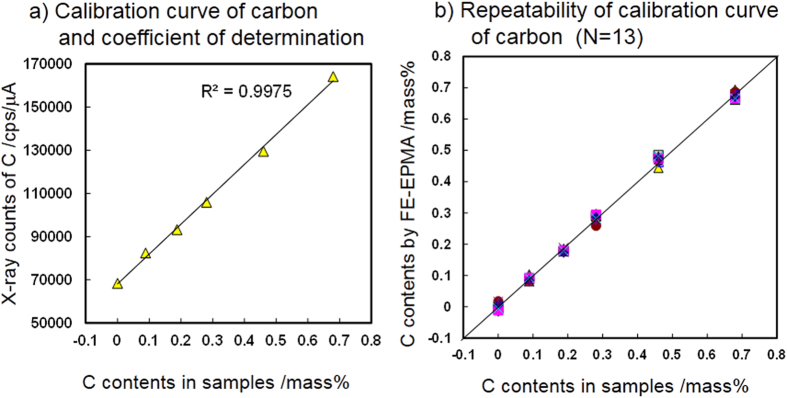
Calibration curve precision and repeatability when using developed instrument. (**a**) Calibration curve of carbon and coefficient of determination. (**b**) Repeatability of calibration curve of carbon (N = 13).

Figure 3a shows one example in which the C–K X-ray intensity(cps/μA) is plotted as a function of the carbon contents in the specimens. R^2^ values ranged from 0.9963 to 0.9985 in all measurements, which indicates good linearity between the X-ray intensity and carbon content. Calibration curves were obtained for each measurement. The standard deviation of the difference between the carbon contents measured by chemical analysis and FE-EPMA for each specimen was evaluated as 0.005 mass%–0.012 mass% for all measurements. Figure 3b shows the quantified carbon content evaluated from the obtained calibration curves by the 13 measurements as a function of the certified carbon contents. Table 1 shows the carbon contents determined by chemical analysis (a), average carbon contents determined by FE-EPMA (b), the difference between the chemical analysis and FE-EPMA results (c) and the standard error of (b) of the 13 measurements (d) for each specimen. These results show not only high accuracy but also very good inter-date repeatability of the point measurements with plasma cleaner in the specimen chamber and liquid nitrogen trap. Under the experimental conditions used in this study, the carbon content at a selected point on a steel sample can be determined with accuracy of better than 0.01 mass% and inter-date repeatability of about 0.01 mass%.

The X-ray count (N) in EPMA measurements has an unavoidable statistical error 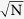. For example, N of the pure iron specimen with the measurement time of 10 s was about 70,000 cps/μA. Due to this statistical error of N, the slope and intercept of the calibration curve also contain error. The error of the carbon content was estimated as about 0.01 mass%, considering the chain rule of the errors of the X-ray count and the slope and intercept of the calibration curve. This value is essentially the same as the inter-date reproducibility mentioned above, indicating that the precision of measurements by our electron microprobe instrument is mainly limited by the statistical error of X-ray counts.

The standard deviation for mapping analysis of the difference between the carbon contents measured by chemical analysis and FE-EPMA for each specimen was 0.008 mass%. This is basically the same value as in the point analysis, because the standard deviation of carbon content is mainly the result of the statistical error of X-ray counts. The systematic error of the quantified values caused by contamination is very low.

**Figure 4:**
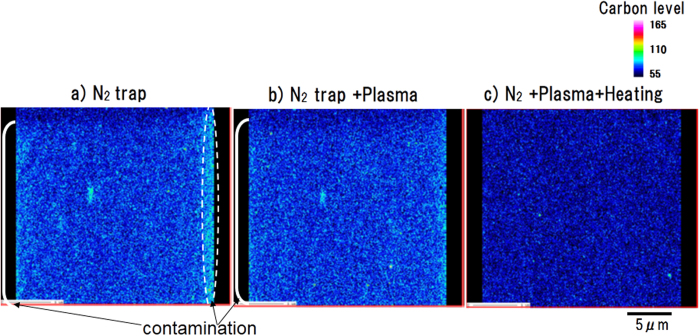
Carbon mapping on pure iron under various combinations of conditions. (**a**) Liquid nitrogen trap alone (**b**) Liquid nitrogen trap plus plasma irradiation (**c**) Liquid nitrogen trap plus plasma irradiation plus sample heating.

Figure 4 shows the mapping data for the carbon intensity(counts) on pure iron when measured under various experimental conditions: liquid nitrogen trap alone, liquid nitrogen trap plus plasma irradiation in the specimen chamber, and liquid nitrogen trap plus plasma irradiation plus sample heating. A focused electron beam was used as the primary electron beam. The mapping measurements were carried out using the step size of 0.08 μm and dwell time of 20 ms. The total measurement time under this experimental condition was about 1300 sec. The X-ray counts from the three detectors were integrated. With the liquid nitrogen trap (Fig. 4a) and plasma irradiation (Fig. 4b), the intensity is low (cyan) at the start of the measurements (upper part of mapping), and gradually increases (parentheses and dotted lines) as the measurements progress. With the liquid nitrogen trap alone, further increases in C–K X-ray intensity are seen on both sides of the mapping. In the measurements with sample heating (Fig. 4c), the carbon X-ray intensity is uniformly low (cyan), indicating that contaminant buildup was satisfactorily suppressed during the electron beam scanning needed for two-dimensional FE-EPMA measurements.

A DP steel (Fe-0.15 mass%C-2.0 mass%Si-1.5 mass%Mn) heated at 750 °C for 150 s in the α + γ two-phase region was measured under the conventional experimental condition (liquid nitrogen trap and one spectrometer) and the optimized condition (liquid nitrogen trap, plasma cleaner, sample heating and three spectrometers) by using the developed instrument. The FE-EPMA measurement conditions were set to an accelerating voltage of 7 kV and beam current of 50 nA, with the beam diameter minimal (focused) in both measurements, and carbon mapping was performed with a 0.17 μm step size and 20 ms dwell time. The mapping intensity data were quantified by the calibration curves obtained for the pure Fe and certified reference steels mentioned above.

**Figure 5:**
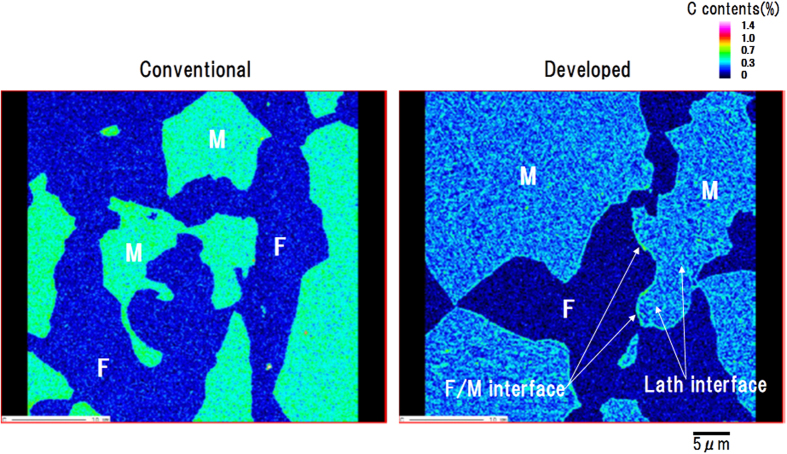
Relationship between carbon mapping of dual-phase steel using conventional instrument and developed instrument.

Figure 5 shows the quantitative carbon map obtained for the DP steel using the conventional instrument (a) and the developed instrument (b). In the mapping by the developed instrument, the carbon concentration in the ferrite phase (F) is extremely low, and a second phase of martensite (M), which transformed from austenite during quenching, is observed. The carbon in the martensite is not uniform, which may be related to the microstructure of martensite^29^. Furthermore, concentrations of carbon are clearly observed at the lath boundaries^30^,^31^,^32^ and interfaces^33^. If the lath boundaries are treated as arrangements of dislocations like low-angle grain boundaries, then this observation can be attributed to segregation of the carbon to the dislocations, as in a Cottrell atmosphere^34^,^35^,^36^. It may also be noted that the concentrations of carbon at the F/M interfaces were not the same across all the interfaces, and local variation was observed. It is well known that carbon atoms can move in an iron matrix at temperatures as low as around 100 °C, but carbon atom movement at 100 °C was negligible in this case.

The new FE-EPMA equipped with the plasma cleaner and heating stage in the specimen chamber was developed to enable more precise microanalysis of carbon in complex steel materials. Our data show that sample heating is a particularly effective method for suppressing carbon contamination buildup during measurements. The FE-EPMA technique developed in this study permitted precise carbon distribution mapping of the steel matrix, as shown in Fig. 5.

## Additional Information

**How to cite this article**: Yamashita, T. *et al*. Novel technique to suppress hydrocarbon contamination for high accuracy determination of carbon content in steel by FE-EPMA. *Sci. Rep.* **6**, 29825; doi: 10.1038/srep29825 (2016).

## Figures and Tables

**Table 1:** Quantization precision according to repeatability using developed instrument.

**(a) chemical analysis**	**(b) EPMA**	**(c) difference between (a) and (b)**	**(d) standard error**
0	0.002	0.002	0.003
0.089	0.089	0	0.003
0.188	0.179	−0.009	0.001
0.281	0.283	0.002	0.003
0.46	0.472	0.012	0.003
0.68	0.674	−0.006	0.003

Mass%, N = 13.
