# Corrigendum: Novel technique to suppress hydrocarbon contamination for high accuracy determination of carbon content in steel by FE-EPMA

**DOI:** 10.1038/srep33940

**Published:** 2016-09-30

**Authors:** Takako Yamashita, Yuji Tanaka, Masayasu Nagoshi, Kiyohito Ishida

Scientific Reports 6: Article number: 2982510.1038/srep29825; published online: 07 19 2016; updated: 09 30 2016

The original version of this Article contained a typographical error in the spelling of the author Masayasu Nagoshi, which was incorrectly given as Masayasu Yagoshi. This has now been corrected in the PDF and HTML versions of the Article.

